# Transgressive Segregation, Reciprocal Effects and Multi-Trait Selection in an F2 Bread Wheat Diallel Under Speed Breeding

**DOI:** 10.3390/plants15182780

**Published:** 2026-09-10

**Authors:** Levent Yorulmaz, Süreyya Betül Rufaioğlu, Murat Tunç, Sibel İpekeşen, Mihriban Okur, Behiye Tuba Biçer, Cuma Akıncı

**Affiliations:** 1 Department of Field Crops, Faculty of Agriculture, Dicle University, Diyarbakır 21280, Türkiye; levent.yorulmaz@dicle.edu.tr (L.Y.); sibel.ipekesen@dicle.edu.tr (S.İ.); tbicer@dicle.edu.tr (B.T.B.); 2 Department of Soil Science and Plant Nutrition, Faculty of Agriculture, Harran University, Şanlıurfa 63300, Türkiye; sureyyarufaioglu@harran.edu.tr; 3 Department of Field Crops, Faculty of Agriculture, Harran University, Şanlıurfa 63300, Türkiye; murattunc@harran.edu.tr; 4 Department of Agricultural Machinery and Technologies Engineering, Faculty of Agriculture, Dicle University, Diyarbakır 21280, Türkiye; mihriban.okur@dicle.edu.tr

**Keywords:** bread wheat, F_2_ population, partial (smart) diallel, reciprocal effects, transgressive segregation, path analysis, canonical discriminant analysis, MGIDI, speed breeding

## Abstract

Speed breeding accelerates wheat line development, yet most reports address protocol optimisation rather than how genetic variation is expressed within the segregating populations grown in the chamber. Because a controlled long-day chamber imposes a uniform, low-stress environment, it minimises the environmental component of variance and lets genetic differences among crosses be observed with little environmental confounding. We characterised an F_2_ population of 851 single plants derived from a partial (“smart”) diallel with reciprocals among three bread wheat cultivars, namely Adana-99 (heat-tolerant), Alada and Lucilla (both heat-sensitive), all four crosses sharing Adana-99 as a common parent. Plants were grown under a protocol-conformant 22 h light/2 h dark photoperiod (light 24.1 °C, dark 17.9 °C) and scored for twelve agro-morphological, phenological and physiological traits. All crosses showed wide phenotypic variation and abundant favourable transgressive segregation; the Adana-99 × Lucilla crosses produced the highest frequencies of transgressive segregants for grain weight per spike, biomass and harvest index, and of early-flowering individuals. Reciprocal differences were significant for biomass, grains per spike and grain weight per spike, in each case favouring Adana-99 as the female parent, indicating a maternal (or cytoplasmic) contribution. After excluding harvest index, which is algebraically derived from grain yield, path analysis identified grain weight per spike and biomass as the direct determinants of single-plant grain yield. Canonical discriminant analysis and a multi-trait genotype–ideotype distance index (MGIDI) were used to describe the multivariate divergence among crosses and to identify segregants combining favourable values across traits. The selected segregants and the Adana-99 × Lucilla combinations are proposed as candidates for multi-environment field validation.

## 1. Introduction

Bread wheat (*Triticum aestivum* L.) is the most widely cultivated cereal in the world and supplies close to one-fifth of the calories and protein in the human diet [1,2]. Sustaining wheat supply under increasingly variable climates requires continued genetic gain in grain yield together with yield stability [3]. Genetic gain depends on the breeder’s ability to generate populations that harbour useful allelic variation, to identify the trait combinations most predictive of yield, and to select efficiently in early generations [4,5].

The F_2_ generation is the earliest segregating generation after hybridisation and carries the greatest amount of additive, dominance and epistatic variance available in any selfing generation [6]. Because individual F_2_ plants are genetically unique, single-plant evaluation at this stage is informative for dissecting trait–trait relationships through path-coefficient analysis [7,8] and, in particular, for identifying transgressive segregants that exceed both parents. Transgressive segregation, the appearance of individuals whose phenotype lies outside the parental range, is a principal source of the novel recombinants on which early-generation selection depends [6]. The F_2_ generation is therefore a conventional starting point for selection in self-pollinating cereals.

Speed breeding, the use of extended photoperiods, typically 20–22 h, under controlled environments, shortens the interval to anthesis and physiological maturity and permits several generations per year [9,10], and has been incorporated into wheat-breeding pipelines to compress the cultivar-development cycle [11,12]. A controlled long-day chamber also imposes a spatially uniform, low-stress environment. This has an analytical consequence that is usually overlooked: by compressing the environmental component of variance, a uniform chamber allows differences among genotypes and crosses to be expressed with little environmental confounding. Phenotypes recorded inside the chamber remain specific to that environment, but the uniformity of the chamber makes it a useful setting in which to compare the standing genetic variation of a segregating population.

Diallel mating designs allow systematic comparison of genotype combinations; partial (incomplete) diallels, in which only a subset of the possible crosses is made, are used where crossing capacity is limited [13,14,15]. When the subset is chosen deliberately rather than at random here, a “smart” partial diallel built around a single common parent and realised in both directions, it concentrates crossing effort on the comparisons of interest and, because reciprocal pairs are included, permits formal testing of maternal and cytoplasmic effects, which are frequently not examined in conventional yield-component studies [16,17].

In this study, three parents were chosen to combine contrasting responses to heat; Adana-99 is a heat-tolerant cultivar of Eastern Mediterranean origin, whereas Alada and Lucilla are heat-sensitive. By placing Adana-99 as the common parent of every cross, and in both the maternal and the paternal role, the design isolates the contribution of this tolerant genotype, including any maternal contribution to progeny performance. The present trial was conducted exclusively under uniform, non-stress speed-breeding conditions; it therefore does not test heat tolerance directly, but evaluates the standing genetic superiority conferred by the tolerant parent in an environment in which environmental variation is minimised.

This study characterises an F_2_ bread wheat partial diallel population involving reciprocal crosses under speed-breeding conditions. It comprehensively evaluates phenotypic variation and differences among crosses and identifies superior segregants using transgressive-segregation profiling, canonical discriminant analysis, and the multi-trait genotype–ideotype distance index (MGIDI). Specifically, we (i) quantify phenotypic variation and favourable transgressive segregation within each of the four crosses, (ii) test for reciprocal effects across the two reciprocal pairs, (iii) decompose trait–yield relationships by correlation and path-coefficient analysis, (iv) describe the multivariate divergence among crosses by principal component analysis, hierarchical clustering and canonical discriminant analysis, and (v) identify multi-trait selections with MGIDI.

## 2. Materials and Methods

### 2.1. Plant Material and Crossing Scheme

Three bread wheat (*Triticum aestivum* L.) cultivars were used as parents: Adana-99 (P1), Alada (P2) and Lucilla (P3). Their origin and main characteristics are given in Table 1.

The parents were crossed following a “smart” diallel approach. In this approach, the parents are chosen on the basis of their response to heat stress, their agronomic performance and their genetic background, and rather than producing every possible combination as in a classical diallel, only the target combinations with the greatest potential to yield superior progeny are made [13,14]. This uses labour, time and growing space more efficiently and, at the same time, increases the probability of recovering superior individuals [14]. Accordingly, the design was built around the heat-tolerant cultivar Adana-99 (P1) as a common parent and realised in both directions with each of the two heat-sensitive cultivars, Alada (P2) and Lucilla (P3), giving four combinations, P1 × P2, P2 × P1, P1 × P3 and P3 × P1, that form two reciprocal pairs. Including the reciprocals allows the maternal and cytoplasmic contribution of the tolerant parent to be tested directly [16,17], which was the specific objective of the design; the P2 × P3/P3 × P2 pair, which does not involve Adana-99, was therefore omitted.

F_1_ plants were self-pollinated to generate F_2_ seed. A total of 851 F_2_ individuals (P1 × P2, *n* = 218; P2 × P1, *n* = 219; P1 × P3, *n* = 207; P3 × P1, *n* = 207), together with six single-plant replicates of each parental cultivar (*n* = 18 parental records), were grown for phenotypic evaluation. Sowing was carried out on 5 December 2024.

Crosses were made in a crossing nursery in which the parents were sown every three days over six sowing dates to synchronise flowering. For each cross, the female parent was emasculated immediately before anthesis (Zadoks 50–55) and hand-pollinated at Zadoks 61–65 [18]. Twenty-four F_1_ seeds were obtained per combination, and every F_2_ plant used in the trial was derived from a selfed F_1_ plant, so that each F_2_ individual represents an independent recombination event.

**Table 1:** Origin and main characteristics of the parental bread wheat cultivars used in the study.

Cultivar (Code)	Growth Habit	Grain Color	Awnedness	Heat-Stress Response	Origin (Institute, Year, Location)
Adana-99 (P1)	Spring	White	Awned	Tolerant	Eastern Mediterranean Agricultural Research Institute, 1999—Adana, Türkiye
Alada (P2)	Facultative	Red	Awnless	Sensitive	Maize Research Institute, 2015— Sakarya, Türkiye
Lucilla (P3)	Facultative	Red	Awned	Sensitive	ProGen Seed Inc., 2017—Hatay, Türkiye

Cultivar origin and registration data are from the Turkish national variety catalogue [19]; the heat-stress classification is from a companion study.

### 2.2. Speed-Breeding Conditions

Plants were grown in a controlled-environment speed-breeding chamber under a 22 h light/2 h dark photoperiod, following the protocols of Watson et al. [9] and Ghosh et al. [10]. Illumination was supplied by a multi-band LED system spanning 450–730 nm. Air temperature and relative humidity were logged throughout the 90-day experimental period (5 December 2024 to 4 March 2025). The set-points followed the recommended speed-breeding regime for wheat: the mean temperature during the 22 h light period was 24.1 ± 1.8 °C and during the 2 h dark period 17.9 ± 0.5 °C, giving a 24 h weighted mean of 23.6 ± 1.7 °C, and mean relative humidity was 50.3 ± 6.3%. Light-period temperature rose gradually over the trial, from 22.8 ± 0.9 °C during the first 30 days to 25.8 ± 1.6 °C during the final 30 days, whereas dark-period temperature remained near-constant (17.7–18.0 °C) and relative humidity fluctuated between 40% and 67% without a directional trend. The maximum light-period daily mean recorded was 28.2 °C; the thermal regime therefore remained within the range recommended for speed breeding in wheat [9,10], and no episode of heat stress occurred. This uniform, protocol-conformant environment was chosen deliberately, so that the differences observed among crosses and reciprocals would reflect genetic rather than environmental variation.

The experiment was conducted with six replications, with each of the four F_2_ populations and three parental genotypes represented by six pots within each replication. Six seeds were sown per pot, resulting in 36 plants per F_2_ population or parental genotype within each replication (6 pots × 6 plants). Accordingly, each replication comprised 42 pots (7 genetic materials × 6 pots), resulting in a total of 252 pots across the entire experiment (42 pots × 6 replications). To minimise potential positional and microenvironmental effects, the pots were randomly arranged within each replication, with randomisation performed independently for each replication.

### 2.3. Trait Measurements

Twelve traits were measured on every F_2_ individual and on every parental replicate. The phenological traits were days to heading (DH) and days to flowering (DF), both recorded from sowing. The physiological traits were SPAD readings of the flag leaf at heading (SPADh) and at flowering (SPADf), taken with a Minolta SPAD-502Plus chlorophyll meter (Konica Minolta, Tokyo, Japan) as the mean of three readings per plant. The morphological traits, recorded on the main spike, were plant height (PH, cm; from ground level to the top of the main spike, excluding awns), spike length (SL, cm), spikelets per spike (SNS), grains per spike (GNS) and grain weight per spike (GWS, g). The yield traits were grain yield per plant (GY, g; total grain weight of all spikes), aboveground biomass per plant (BM, g; oven-dried at 70 °C to constant weight) and harvest index (HI, %; HI = 100 × GY/BM).

Grain yield per plant was the combined grain weight of all spikes borne by a plant (main spike plus all tillers). Aboveground biomass was the total dry weight of the shoot measured on a precision balance (0.001 g); spikes were then threshed individually with a single-spike thresher, and the main-spike grain weight and the total grain yield were weighed to 0.001 g.

### 2.4. Statistical Analyses

Analyses were carried out in Python 3.12 with pandas, NumPy, SciPy [20], statsmodels, scikit-learn [21] and NetworkX [22]; the MGIDI index was computed following Olivoto and Nardino [23].

#### 2.4.1. Descriptive Statistics and Transgressive Segregation

Mean, standard deviation, minimum, maximum and the coefficient of variation (CV%) were computed for each trait, pooled across the F_2_ population and within each cross. For every cross and trait, the lower-parent and higher-parent means were used to count segregants transgressing the parental range. Transgression in the favourable direction was defined as exceeding the higher parent for the physiological, spike and yield traits, and as falling below the lower parent (i.e., earlier or shorter) for days to heading, days to flowering and plant height; the number and percentage of favourable transgressive individuals were recorded for each cross.

#### 2.4.2. Cross Effects and Reciprocal Differences

One-way ANOVA followed by Tukey’s HSD post hoc test (α = 0.05) was used to compare the four crosses for each trait. Because twelve traits were tested, the cross effect was additionally evaluated after Bonferroni and Benjamini–Hochberg (false discovery rate) correction; all twelve traits remained significant under FDR, and all except SPAD at flowering and plant height remained significant under the conservative Bonferroni correction. Reciprocal differences were tested within each of the two reciprocal pairs (P1 × P2 vs. P2 × P1; P1 × P3 vs. P3 × P1) using Welch’s *t*-test for unequal variances.

#### 2.4.3. Trait Associations and Path Analysis

Pearson correlation coefficients with two-tailed *p*-values were computed pairwise. Path-coefficient analysis [7,8] was carried out with grain yield per plant as the dependent variable and the remaining eleven traits as predictors; direct effects were obtained by solving the normal equations, the indirect effect of trait *i* via trait *j* as the product of the corresponding correlation and direct effect, and the residual from the unexplained variance.

#### 2.4.4. Multivariate Analyses

Principal component analysis was performed on the standardised (mean = 0, SD = 1) trait matrix, and the first three components were retained. Ward’s hierarchical clustering [24] on Euclidean distances was applied to the same matrix, and four clusters were extracted. A trait correlation network was drawn with NetworkX [22] using a force-directed (Kamada–Kawai) layout [25]. Canonical discriminant analysis (CDA) [26] was performed with the four crosses as the grouping factor to describe the between-cross divergence: standardised canonical coefficients, the proportion of between-cross variance explained by each canonical function and the position of the cross centroids were obtained, and squared Mahalanobis distances between centroids were calculated.

#### 2.4.5. Multi-Trait Selection

The multi-trait genotype–ideotype distance index (MGIDI) of Olivoto and Nardino [23] was used to identify segregants combining favourable values across the twelve traits. Each trait was first rescaled to a 0–100 range in the desired direction (increase for the yield, spike and physiological traits; decrease earliness and shorter stature for days to heading, days to flowering and plant height); the rescaled traits were reduced by factor analysis to account for multicollinearity; an ideotype was defined as the vector of optimal rescaled values; and the MGIDI of each individual was computed as the Euclidean distance, in factor-score space, between the individual and the ideotype, so that lower values denote individuals closer to the ideotype. A selection intensity of 15% was applied, and the strengths and weaknesses of the selected group were summarised by the contribution of each retained factor. For comparison, direct truncation selection of the top 5% for grain yield was also applied.

## 3. Results

### 3.1. Phenotypic Variation and Parental Performance

The three parents differed for all twelve traits (Table 2). Alada (P2) was the latest parent (DF = 82.0 ± 1.7 d) and had the lowest grain weight per spike (0.35 ± 0.08 g) and the lowest harvest index (12.74 ± 3.82%). Adana-99 (P1) and Lucilla (P3) flowered at a similar time (74.7 ± 2.2 d and 74.2 ± 1.9 d). Adana-99 produced the highest grain yield per plant (0.68 ± 0.27 g) and, with Lucilla, the highest harvest index (16.91 ± 4.49% and 16.97 ± 2.85%), whereas Lucilla had the lowest biomass (2.76 ± 0.37 g).

Because each parental mean rests on only six single plants, the 95% confidence intervals are relatively wide (for Adana-99, for example, ±1.3 d for days to heading and ±0.15 g for grain weight per spike). This uncertainty propagates to the transgressive-segregation thresholds, which are derived from the parental means, so the frequencies reported in Section 3.3 are best regarded as approximate.

**Figure 1:**
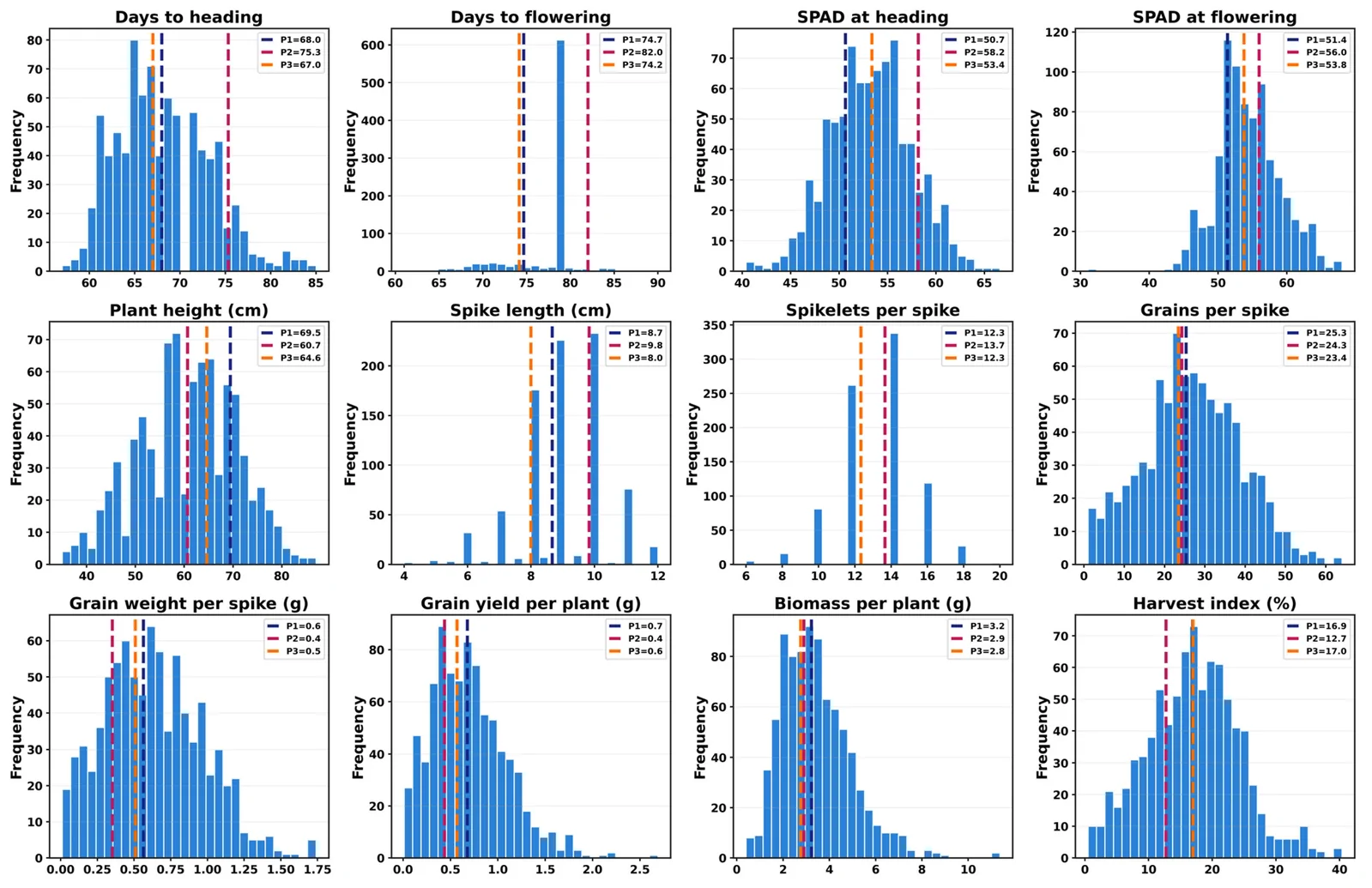
Phenotypic distributions of the twelve traits in the F_2_ population. Dashed vertical lines mark the means of the parents Adana-99 (P1), Alada (P2) and Lucilla (P3). *n* = 851 F_2_ individuals; each parental mean is based on six single plants.

Pooled across the 851 F_2_ individuals, phenotypic variation was wide. Coefficients of variation exceeded 40% for grain yield per plant (57.0%), grain weight per spike (52.8%), grains per spike (44.5%), biomass (43.8%) and harvest index (42.4%). The frequency distributions (Figure 1) were approximately symmetrical for plant height, the SPAD readings and the spike traits, but right-skewed for the yield components, with a tail of individuals well above the better-parent mean. For eleven of the twelve traits, the F_2_ range encompassed and exceeded both parental means, indicating transgressive segregation.

### 3.2. Differences Among Crosses and Reciprocal Effects

One-way ANOVA detected significant differences among the four crosses for eleven of the twelve traits at *p* < 0.001; SPAD at flowering was the exception (*p* = 0.034) (Table 3). The two Adana-99 × Lucilla combinations (P1 × P3 and P3 × P1) had the highest mean grain yield per plant (0.82 and 0.77 g, group a), the highest grain weight per spike (0.76 and 0.71 g, group a) and the highest harvest index (18.21% and 19.55%, group a), and were separated from the two Adana-99 × Alada combinations by Tukey’s HSD for these traits.

**Figure 2:**
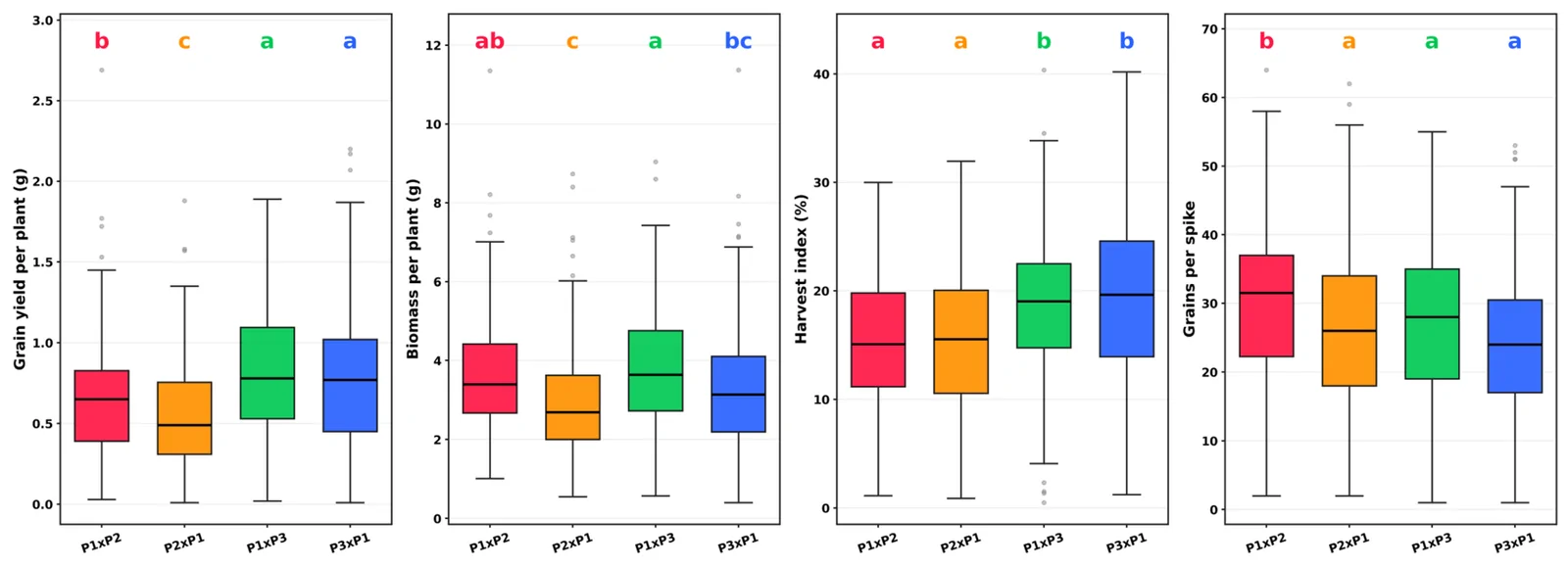
Distribution of four yield-related traits across the four crosses. Letters above each box denote homogeneous groups according to Tukey’s HSD (α = 0.05); *n* = 218 (P1 × P2), 219 (P2 × P1), 207 (P1 × P3) and 207 (P3 × P1).

Welch tests within the two reciprocal pairs detected significant differences for several traits (Table 4). Biomass differed significantly in both pairs: with Adana-99 as the female parent, the combination produced 23% more biomass than its reciprocal in the P1 × P2/P2 × P1 pair (3.64 vs. 2.96 g; *p* < 0.001), and the same direction was seen in the P1 × P3/P3 × P1 pair (3.79 vs. 3.29 g; *p* < 0.001). Grains per spike differed significantly in both pairs, again favouring Adana-99 as the female parent. Grain weight per spike and grain yield per plant differed significantly in the P1 × P2/P2 × P1 pair, and plant height in the P1 × P3/P3 × P1 pair. Among-cross and reciprocal comparisons of phenology are based on days to heading; days to flowering, for which the P1 × P3 cross has a single recorded value, is reported descriptively. Days to heading differed only slightly between reciprocals in both pairs (Figure 2).

### 3.3. Transgressive Segregation

For every cross, the F_2_ minima and maxima extended beyond the parental means for almost all traits, confirming that recombination in the F_2_ generated wide phenotypic distributions and a substantial pool of transgressive segregants (Table 5). The four crosses differed markedly in where these transgressive individuals were concentrated.

In the P1 × P2 cross (Adana-99 × Alada), variation was greatest in the yield components, and favourable transgressive individuals were most frequent for spikelets per spike (71.1%), grains per spike (66.1%) and biomass (56.0%), identifying this combination as a promising source for spike fertility and biomass accumulation. Its reciprocal, P2 × P1, retained high variation for the spike and physiological traits (favourable transgressants of 69.4% for spikelets per spike and 41.7% for SPAD at flowering) but yielded fewer transgressive individuals for grain yield (32.0%) and grain weight per spike (36.1%), so that superior grain yield was concentrated in a smaller number of plants an early indication that the direction of the cross influences the expression of some yield components.

The Adana-99 × Lucilla crosses (P1 × P3 and P3 × P1) were the richest in favourable transgressive segregants for both earliness and yield. In P1 × P3, 73.4% and 76.3% of individuals were earlier than the earlier parent for days to heading and days to flowering, and 72.0%, 63.3%, 63.3% and 57.5% were favourable transgressants for grain weight per spike, biomass, harvest index and grain yield. The reciprocal P3 × P1 showed a similar profile (63.3% and 70.1% early transgressants for days to heading and days to flowering; 63.3%, 63.3% and 62.8% favourable transgressants for grain weight per spike, grain yield and harvest index). The co-occurrence of early maturity and high yield potential in the same cross is an advantage under speed breeding, where rapid generation turnover and effective selection can be combined. The differences between P1 × P3 and P3 × P1 for individual traits are consistent with the reciprocal effects detected in Section 3.2.

### 3.4. Trait Correlations and Path Analysis

**Figure 3:**
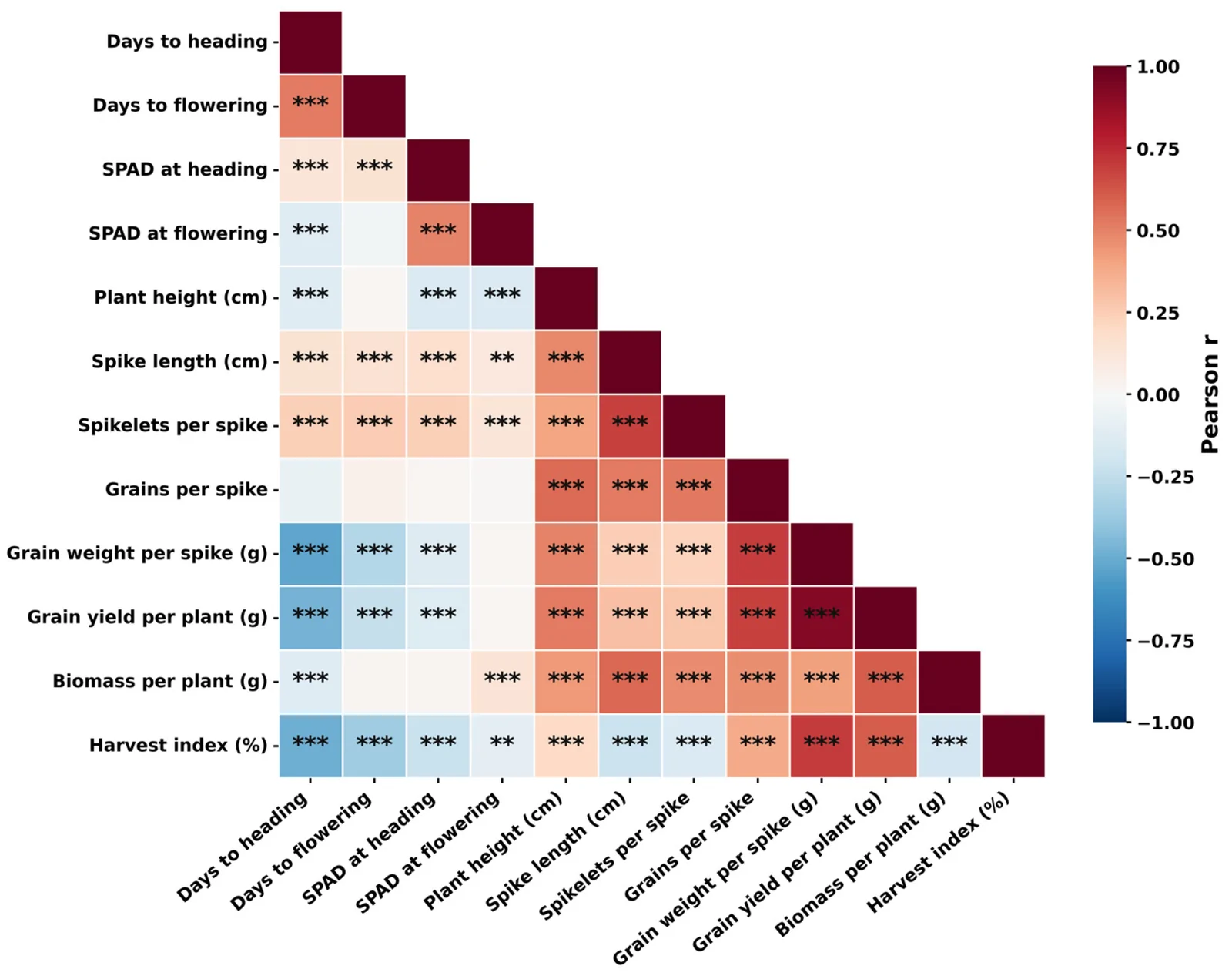
Pearson correlation heat-map for the twelve traits in the F_2_ population. ** *p* < 0.01; *** *p* < 0.05.

Grain yield per plant was most closely associated with grain weight per spike (r = 0.92, *p* < 0.001), followed by grains per spike (r = 0.68), biomass (r = 0.61), harvest index (r = 0.60) and plant height (r = 0.52) (Figure 3). Days to heading was negatively correlated with grain yield (r = −0.47, *p* < 0.001), indicating that earlier-heading individuals tended to out-yield later ones under these conditions. The SPAD readings showed weak associations with the yield components (|r| < 0.15).

**Figure 4:**
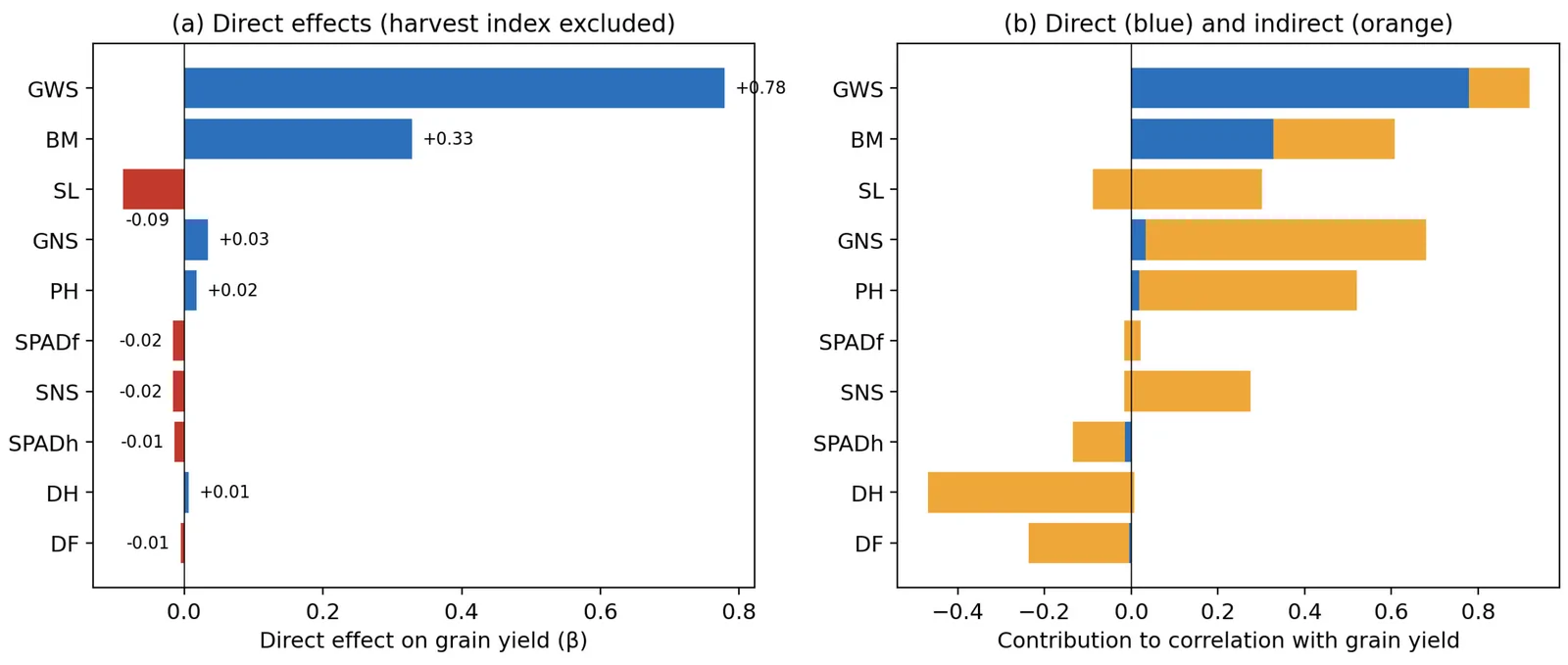
Path-coefficient decomposition for grain yield per plant. (**a**) Direct effects (β), sorted by absolute magnitude; (**b**) decomposition of each predictor’s correlation with grain yield into its direct and total indirect components.

Because harvest index is defined as 100 × GY/BM and is therefore an algebraic function of grain yield, it was excluded from the path model to avoid circularity. With the remaining ten traits as predictors, the analysis gave a residual of 0.292, so that 91.4% of the variation in F_2_ grain yield was accounted for. Two traits had substantial positive direct effects, namely grain weight per spike (β = 0.78) and biomass (β = 0.33), while all other direct effects were below 0.10 in absolute value (Table 6, Figure 4a). Grains per spike (r = 0.68) and plant height (r = 0.52), although correlated with grain yield, transmitted almost their entire effect indirectly, largely through grain weight per spike and biomass (Figure 4b). Because grain weight per spike is very strongly correlated with grain yield (r = 0.92), its large direct coefficient partly reflects this near collinearity.

### 3.5. Multivariate Structure: PCA and Clustering

**Figure 5:**
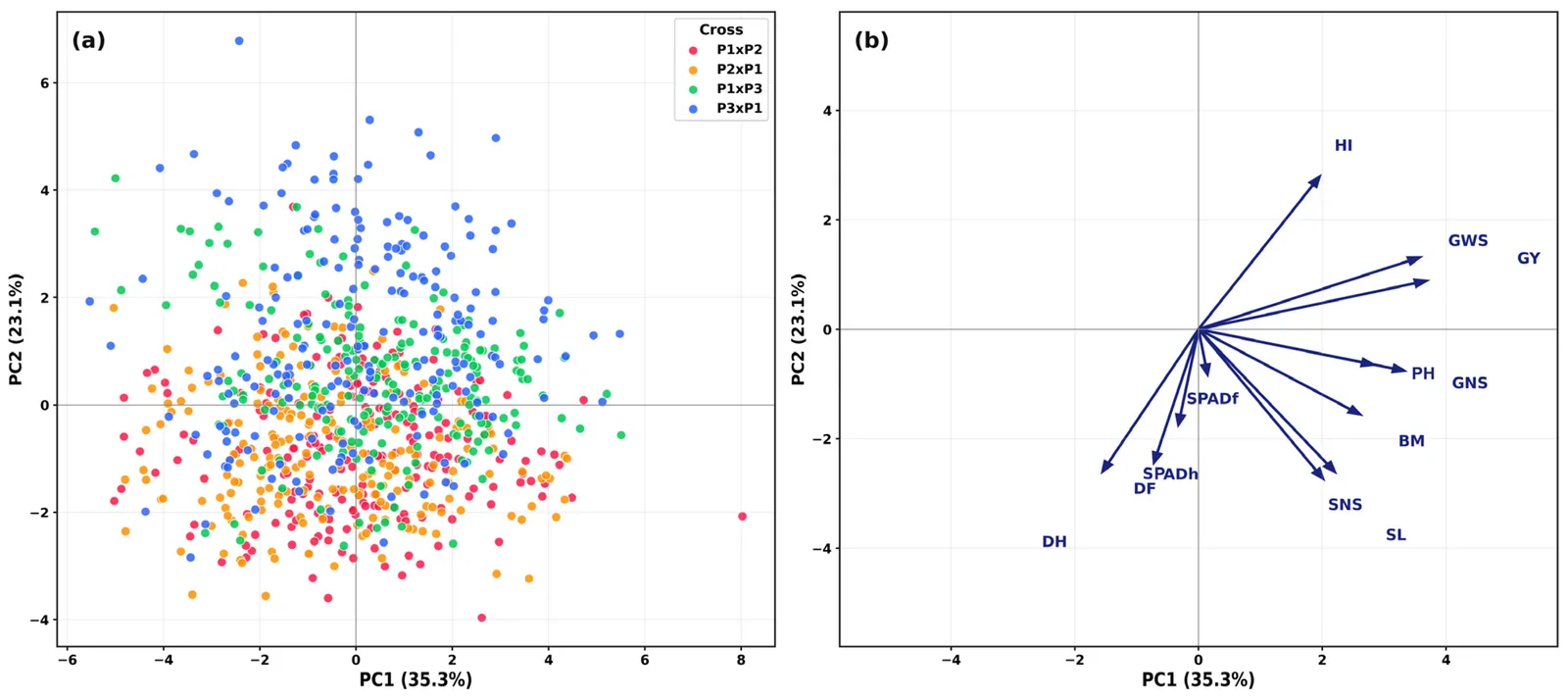
Principal component analysis. (**a**) Score plot of the F_2_ individuals on PC1 and PC2, coloured by cross; (**b**) loading vectors of the twelve traits on PC1 and PC2.

The first three principal components accounted for 67.9% of the total trait variation. PC1 (35.3%) loaded positively on biomass, plant height, grains per spike, grain weight per spike and grain yield, describing a general productivity axis; PC2 (23.1%) loaded positively on harvest index and negatively on the phenology traits and SPAD readings, describing an earliness-by-partitioning axis (Figure 5b). The four crosses occupied overlapping but partially distinct regions of the score plot, with P1 × P3 and P3 × P1 individuals displaced towards higher PC1 values (Figure 5a).

**Figure 6:**
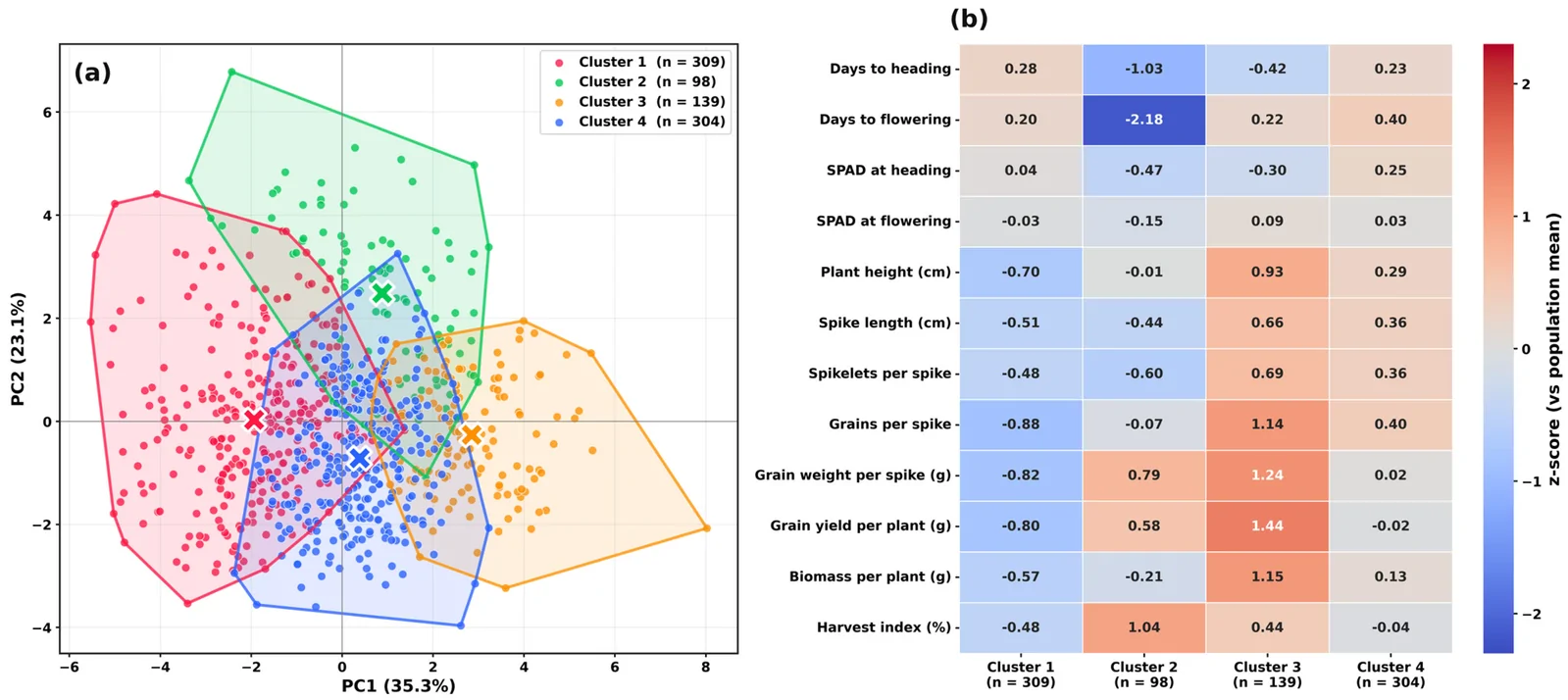
Cluster structure from Ward’s hierarchical clustering on the standardised trait matrix. (**a**) Projection of the four clusters onto the first two principal components; (**b**) heat-map of cluster centroids expressed as z-scores relative to the population mean.

Ward clustering resolved four groups occupying distinct regions of the PC1–PC2 plane (Figure 6a). Cluster 3 (*n* = 139) was the high-yielding group, with z-scores above +0.9 for plant height, grains per spike, grain weight per spike, grain yield and biomass. Cluster 2 (*n* = 98) combined early flowering with a high harvest index (HI z = +1.04; DF z = −2.18). Cluster 1 (*n* = 309) was the lowest-performing group across the yield components, and Cluster 4 (*n* = 305) was intermediate. The population thus contains two distinct high-performing profiles: one based on high biomass and grain weight, and one based on early maturity and efficient partitioning (Figure 6b).

### 3.6. Trait Correlation Network

**Figure 7:**
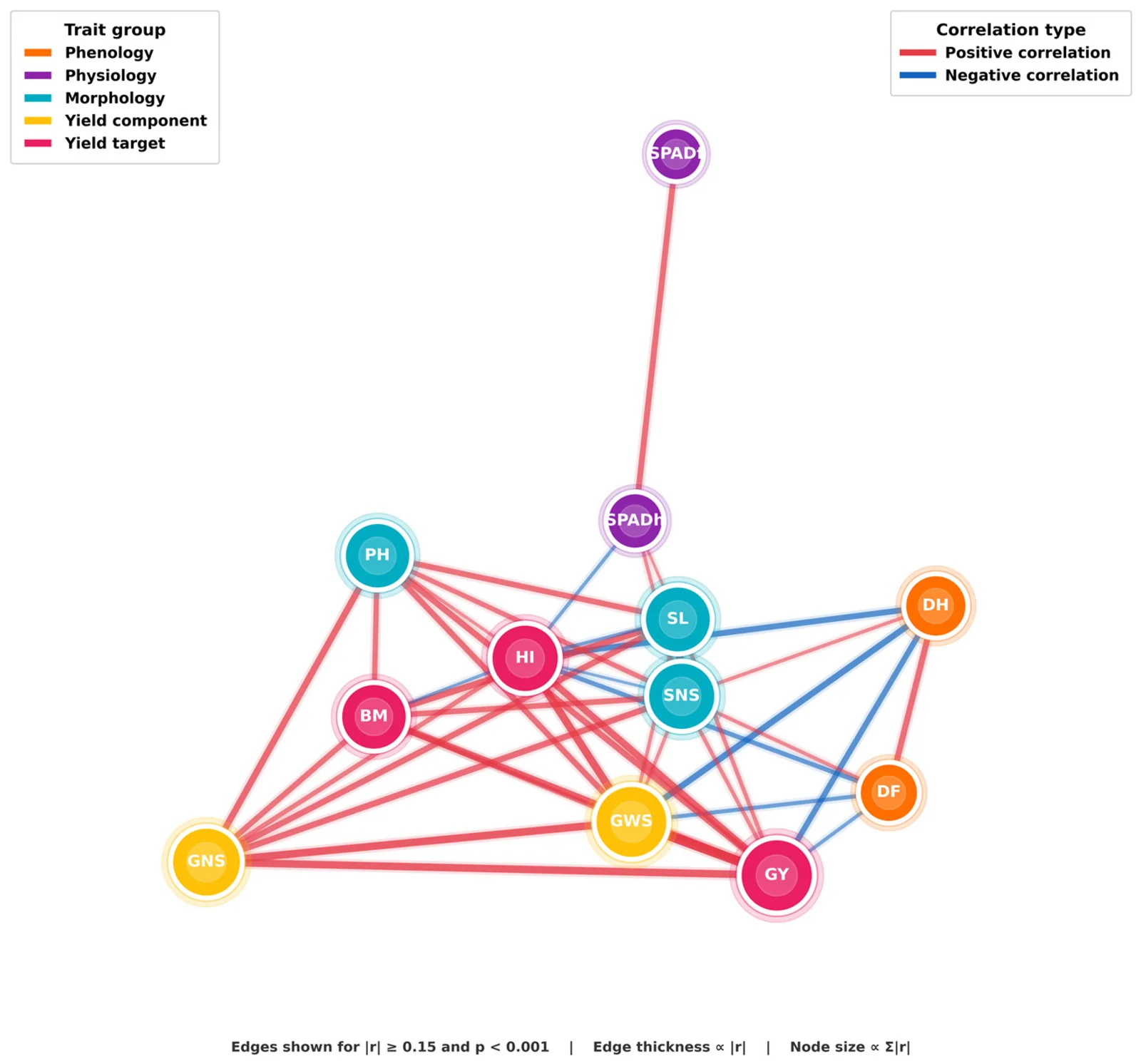
Trait correlation network. Nodes represent the twelve traits, coloured by functional group; node size is proportional to the sum of the absolute correlations of the trait. Edges represent pairwise Pearson correlations with |r| ≥ 0.15 and *p* < 0.001; edge thickness is proportional to |r| and edge colour indicates the sign.

The correlation network (Figure 7) showed an interconnected yield module comprising grain yield, grain weight per spike, biomass, grains per spike, harvest index and plant height, joined by positive correlations, and linked to the spike traits by moderate positive correlations and to the phenology traits by negative correlations. The SPAD readings occupied a peripheral position, largely independent of the yield architecture, which is consistent with the near-zero direct path coefficients estimated for the same traits (Table 6).

### 3.7. Canonical Discriminant Analysis

**Figure 8:**
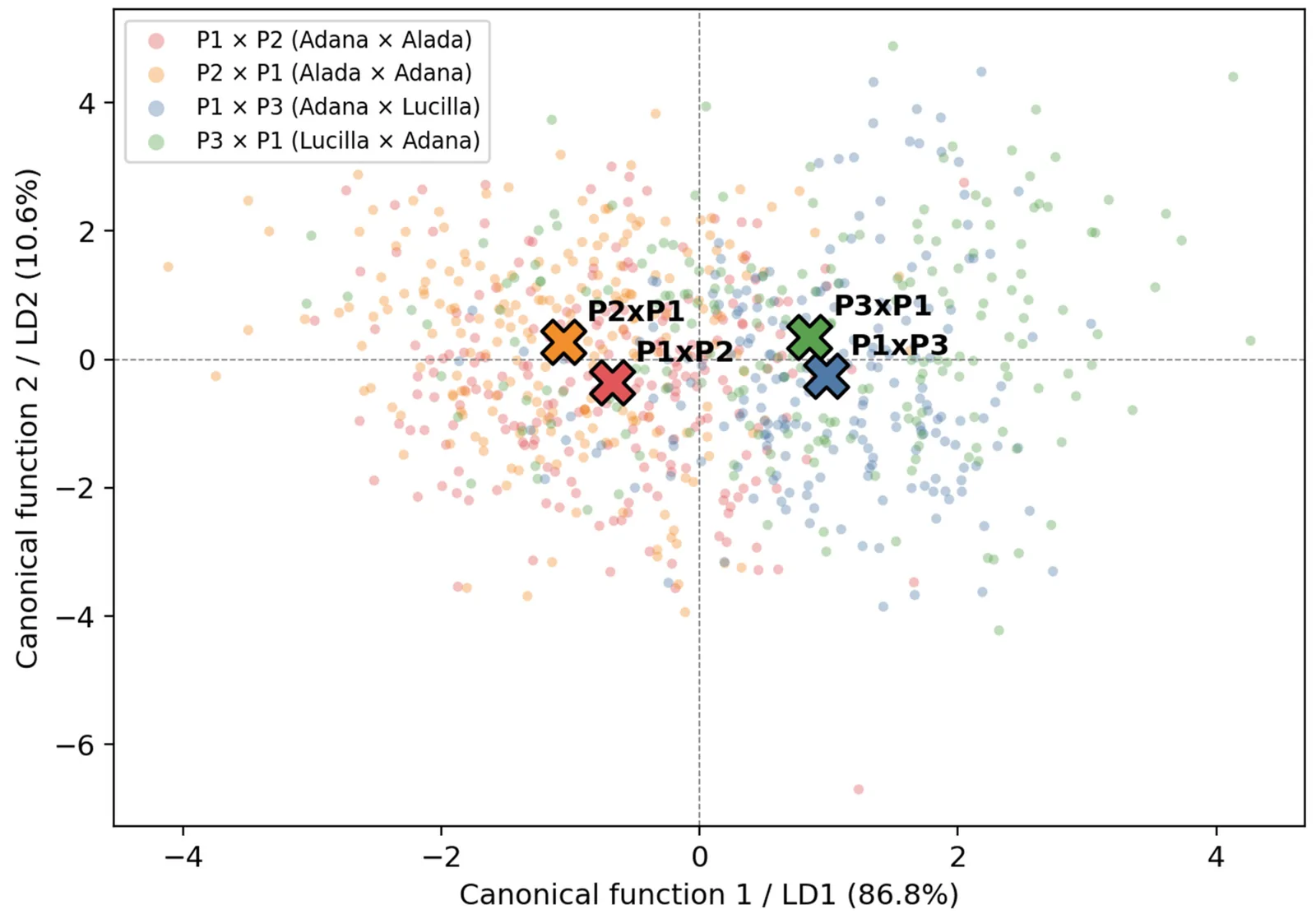
Canonical discriminant analysis of the four crosses on the first two canonical functions (LD1, 86.8%; LD2, 10.6%).

Canonical discriminant analysis, with the four crosses as the grouping factor and the twelve traits as discriminating variables, concentrated the between-cross divergence on two functions: the first canonical function (LD1) accounted for 86.8% and the second (LD2) for 10.6% of the divergence, 97.4% in total (Figure 8). The structure matrix (Table 7) shows that LD1 was defined by an opposition between the phenological and physiological traits days to heading (−0.74), days to flowering (−0.68) and SPAD at heading (−0.53) and the yield-related traits grain weight per spike (+0.45), grain yield (+0.39) and harvest index (+0.37); LD2 was defined mainly by biomass (−0.62), grains per spike (−0.50) and plant height (−0.37).

On LD1, the two Adana-99 × Alada crosses had negative centroids (P1 × P2 = −0.68, P2 × P1 = −1.06) and the two Adana-99 × Lucilla crosses positive centroids (P1 × P3 = +0.98, P3 × P1 = +0.85), so the primary axis separated the population by cross parentage, combining earliness and yield potential. LD2 separated the reciprocals within each pair: the combinations with Adana-99 as the female parent (P1 × P2, P1 × P3) had negative LD2 scores and their reciprocals (P2 × P1, P3 × P1) positive scores, consistent with the maternal biomass effect described above. Reflecting the segregating nature of the F_2_, the crosses overlapped substantially, and the overall leave-one-out cross-validated correct-classification rate was moderate (54.2%), highest for P2 × P1 (142 of 219 individuals correctly assigned). Canonical discriminant analysis therefore recovered the same phenology-versus-yield and reciprocal structure identified by the univariate, correlation and principal component analyses, and its results are best interpreted together with the transgressive-segregation and MGIDI analyses rather than as a stand-alone classifier.

### 3.8. Multi-Trait Selection (MGIDI)

Direct truncation selection of the top 5% for grain yield identified 43 F_2_ individuals with a mean grain yield of 1.67 g per plant against a population mean of 0.70 g. The Adana-99 × Lucilla crosses P1 × P3 (*n* = 19) and P3 × P1 (*n* = 14) together contributed 33 of the 43 elite individuals (76.7%), against 7 from P1 × P2 (16.3%) and 3 from P2 × P1 (7.0%), confirming this reciprocal pair as the main source of high-yielding segregants.

Because the index was computed from raw single-plant phenotypic values rather than from genotypic estimates, MGIDI is used here as a multi-trait selection heuristic rather than as a genotypic-value index. Because single-trait truncation ignores the correlated architecture of yield, the multi-trait genotype–ideotype distance index (MGIDI) was used to select segregants that combine favourable values across all twelve traits while avoiding the multicollinearity and arbitrary weighting that destabilise classical linear indexes [23]. Factor analysis grouped the twelve traits into three factors (eigenvalue > 1): Factor 1 (days to heading, days to flowering, grain weight per spike, grain yield and harvest index), Factor 2 (plant height, spike length, spikelets per spike, grains per spike and biomass) and Factor 3 (the two SPAD readings).

**Figure 9:**
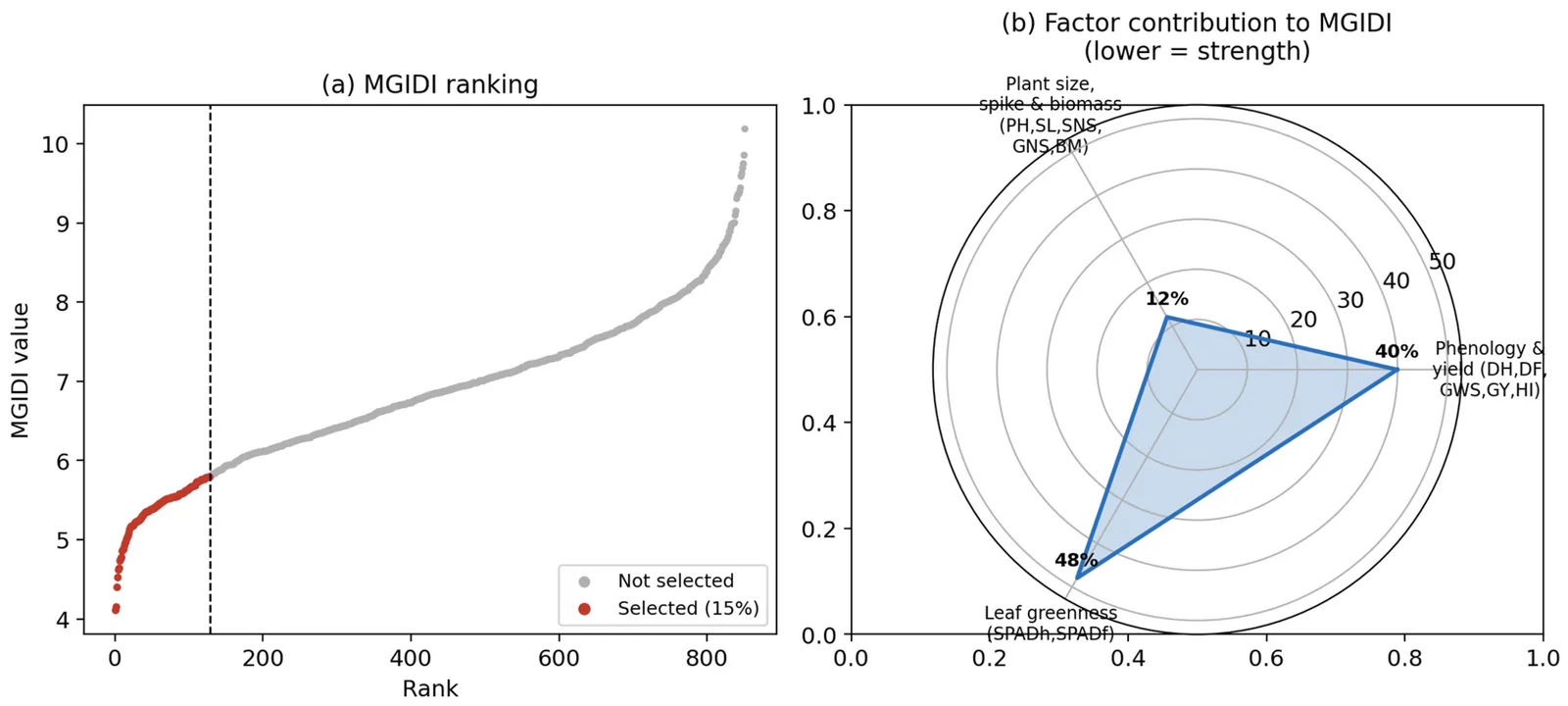
MGIDI multi-trait selection of the F_2_ population. (**a**) MGIDI value of each individual; the dashed line marks the 15% selection threshold, with selected individuals in red. (**b**) Contribution of each of the three factors to the MGIDI distance of the selected group.

Applying MGIDI at a 15% selection intensity retained 128 F_2_ individuals. The Adana-99 × Lucilla crosses again predominated (P3 × P1, *n* = 40; P1 × P3, *n* = 36; together 59.4% of the selection), followed by the Adana-99 × Alada crosses (P2 × P1, *n* = 29; P1 × P2, *n* = 23). Relative to the whole population, the selected group showed favourable selection differentials for every yield-related trait, namely grain yield per plant +51.4%, grain weight per spike +47.1%, biomass +27.6%, grains per spike +25.4% and harvest index +17.7%, together with earlier heading (−5.6%) and flowering (−2.1%) (Table 8). The strengths-and-weaknesses profile (Figure 9b) shows that the selected group lay closest to the ideotype on the plant-size, spike and biomass factor (Factor 2, which contributed only 12.1% of the MGIDI distance), whereas the SPAD factor (Factor 3, 48.0%) was the main weakness, reflecting the weak association of leaf greenness with yield in this population. The multi-trait selection was therefore somewhat more balanced across crosses than the single-trait truncation, but confirmed the same reciprocal pair as the principal source of superior segregants.

## 4. Discussion

The most consistent result of this study was the directional advantage of crosses in which Adana-99 served as the female parent. Because Adana-99 was common to all four crosses, the repeatable superiority of its maternal progeny for biomass, grains per spike and grain weight per spike is most parsimoniously attributed to a maternal contribution of this cultivar rather than to a cytoplasmic effect specific to Alada or Lucilla. Reciprocal and maternal effects on yield components are increasingly reported in wheat, where they act through both cytoplasmic genomes and maternally provisioned resources [27,28]. The present design cannot separate these routes—F_1_ plants were not phenotyped, and seed weight at sowing was not recorded, so a maternal seed-size effect cannot be excluded [16,17]—yet the breeding implication is clear: the choice of seed parent is not neutral, and using Adana-99 as the female consistently produced superior early-generation progeny.

Detecting these effects depended on evaluating the population under uniform speed-breeding conditions. A controlled long-day chamber compresses the environmental component of phenotypic variance, so that among-cross and reciprocal differences are expressed against a low-noise background and are more readily interpreted as genetic than the same contrasts measured in a heterogeneous field. Phenotypes recorded in the chamber remain specific to that environment, and the modest (~3 °C) seasonal rise in light-period temperature makes the phenological values, in particular, regime-specific; because this rise affected all plants alike, it does not bias the comparisons among crosses. Speed breeding thus served here not only to compress the breeding cycle but also as a setting in which the standing genetic variation of a segregating population could be resolved.

Among the phenological traits, days to heading was the more informative descriptor and was negatively correlated with grain yield, so that earlier-heading segregants tended to be the higher yielding under these conditions. Earliness and yield potential were therefore not antagonistic in this material, which is favourable where rapid generation turnover is combined with selection for yield.

The transgressive-segregation profiles translate this variation into breeding terms. Favourable transgressive segregants were abundant in every cross, in keeping with the F_2_ being the generation of maximum segregation and the stage at which parent-exceeding recombinants are most efficiently recovered [29,30,31,32]. Their distribution was cross-specific: the Adana-99 × Lucilla crosses were the richest source of segregants combining earliness with high grain weight per spike, biomass and harvest index, whereas the Adana-99 × Alada crosses were superior for spike fertility. Directing crossing and selection effort according to these complementary strengths is more efficient than treating the crosses as interchangeable.

Correlation and path analysis identified grain weight per spike and biomass as the direct determinants of single-plant grain yield, whereas grains per spike, despite a strong phenotypic correlation with yield, acted almost entirely through indirect channels. This source–sink decomposition indicates that simultaneous selection on grain weight per spike and biomass is more effective than selection on grains per spike alone [33,34]. Because grain weight per spike is nearly collinear with grain yield, it largely carries the yield signal; the multivariate analyses were coherent with this structure, the population being organised along a biomass-driven productivity axis and an earliness-by-partitioning axis, with two high-performing profiles resolved by clustering and the crosses separated along the same directions by canonical discriminant analysis.

For simultaneous selection across traits, the multi-trait genotype–ideotype distance index (MGIDI) was preferred to the classical Smith–Hazel index. In an unreplicated single-plant F_2_, genotypic covariances cannot be estimated directly, and grain yield is nearly collinear with grain weight per spike, conditions under which a linear selection index becomes ill-conditioned and its weights unstable. Being based on factor analysis and on the distance to a predefined ideotype, MGIDI accommodates multicollinearity and is applicable to unreplicated data [23]; it identified balanced segregants that single-trait truncation would have overlooked, while confirming the Adana-99 × Lucilla pair as the principal source of superior individuals.

These conclusions are bounded by the design. A single, unreplicated speed-breeding environment does not permit estimation of genotype × environment interaction; no stress was imposed, and the phenotypes are not yet anchored to molecular markers. The segregants and crosses identified here are accordingly best regarded as hypotheses for testing: the immediate priorities are to advance the selections to replicated, multi-environment field trials and to map the underlying loci in derived F_3_/F_4_ lines, which would establish the stability and the genetic basis of the superior phenotypes observed under speed breeding.

## 5. Conclusions

An F_2_ bread wheat population of 851 single plants, derived from a partial (smart) diallel with reciprocals around a heat-tolerant common parent and phenotyped entirely under uniform speed-breeding conditions, showed wide phenotypic variation and abundant favourable transgressive segregation. Reciprocal differences for biomass, grains per spike and grain weight per spike consistently favoured Adana-99 as the female parent, indicating that the choice of seed parent is not neutral in this material. Path analysis identified grain weight per spike and biomass as the direct determinants of single-plant grain yield, and canonical discriminant analysis and the MGIDI index were used to describe the divergence among crosses and to select balanced segregants. The Adana-99 × Lucilla combinations contributed the large majority of the high-yielding segregants and combined earliness with yield potential. By evaluating the population in a uniform, non-stress chamber, the study isolates the genetic and maternal contribution of the tolerant parent with minimal environmental confounding; the selections and crosses identified are proposed as candidates for multi-environment field validation and, subsequently, for QTL mapping in derived F3/F4 lines.

## Figures and Tables

**Table 2:** Mean ± standard deviation of the three parental cultivars for the twelve traits. Within a row, means followed by the same letter do not differ at *p* = 0.05 (Tukey’s HSD).

Trait	Adana-99 (P1)	Alada (P2)	Lucilla (P3)
Days to heading (d)	68.00 ± 1.26 b	75.33 ± 1.63 a	67.00 ± 3.46 b
Days to flowering (d)	74.67 ± 2.16 b	82.00 ± 1.67 a	74.17 ± 1.94 b
SPAD at heading	50.67 ± 0.60 b	58.18 ± 0.43 a	53.40 ± 3.82 b
SPAD at flowering	51.38 ± 1.04 b	55.98 ± 1.41 a	53.77 ± 3.99 a
Plant height (cm)	69.45 ± 6.02 a	60.68 ± 2.31 b	64.62 ± 3.17 a
Spike length (cm)	8.67 ± 0.52 b	9.83 ± 0.41 a	8.00 ± 0.89 b
Spikelets per spike	12.33 ± 0.82 a	13.67 ± 0.82 a	12.33 ± 1.51 a
Grains per spike	25.33 ± 4.00 a	24.28 ± 4.42 a	23.44 ± 2.46 a
Grain weight per spike (g)	0.56 ± 0.14 a	0.35 ± 0.08 b	0.51 ± 0.12 a
Grain yield per plant (g)	0.68 ± 0.27 a	0.44 ± 0.21 a	0.57 ± 0.13 a
Biomass per plant (g)	3.22 ± 0.42 a	2.90 ± 0.51 a	2.76 ± 0.37 a
Harvest index (%)	16.91 ± 4.49 a	12.74 ± 3.82 a	16.97 ± 2.85 a

**Table 3:** Mean values of the four crosses for the twelve traits, with one-way ANOVA. Within a row, means followed by the same letter do not differ at *p* = 0.05 (Tukey’s HSD).

Trait	P1 × P2	P2 × P1	P1 × P3	P3 × P1	F	*p*-Value
Days to heading	70.24 a	70.88 a	64.97 b	65.86 b	95.00	5.29 × 10^−53^
Days to flowering	78.92 a	79.42 a	79.00 a	72.71 b	300.43	9.54 × 10^−133^
SPAD at heading	54.05 b	55.33 a	51.87 c	51.80 c	41.95	2.81 × 10^−25^
SPAD at flowering	54.48 a	55.15 a	54.06 a	54.00 b	2.90	0.034
Plant height (cm)	61.98 a	60.17 a	61.40 a	58.92 b	3.97	0.008
Spike length (cm)	9.35 a	9.21 a	8.78 b	8.68 b	12.46	5.63 × 10^−8^
Spikelets per spike	14.04 a	13.85 a	12.79 b	12.31 b	37.39	1.07 × 10^−22^
Grains per spike	29.97 a	26.33 b	26.82 b	23.94 b	9.49	3.58 × 10^−6^
Grain weight per spike (g)	0.58 b	0.50 c	0.76 a	0.71 a	29.51	3.74 × 10^−18^
Grain yield per plant (g)	0.65 b	0.55 c	0.82 a	0.77 a	21.72	1.47 × 10^−13^
Biomass per plant (g)	3.64 a	2.96 b	3.79 a	3.29 b	13.97	6.75 × 10^−9^
Harvest index (%)	15.01 b	15.37 b	18.21 a	19.55 a	21.31	2.60 × 10^−13^

All F-values are significant at *p* < 0.05 (SPAD at flowering) or *p* < 0.001 (all other traits). P1 = Adana-99, P2 = Alada, P3 = Lucilla. For the P1 × P3 cross, days to flowering corresponds to a single recorded value.

**Table 4:** Welch *t*-tests for reciprocal differences within the two reciprocal pairs. Mean (D), direct cross; Mean (R), reciprocal cross.

Trait	Direct	Mean (D)	Reciprocal	Mean (R)	Diff.	t	*p*-Value	Sig.
Days to heading	P1 × P2	70.24	P2 × P1	70.88	−0.64	−1.47	0.143	ns
Days to heading	P1 × P3	64.97	P3 × P1	65.86	−0.89	−2.04	0.042	*
Days to flowering	P1 × P2	78.92	P2 × P1	79.42	−0.50	−3.66	<0.001	***
Days to flowering	P1 × P3	79.00	P3 × P1	72.71	6.29	18.02	<0.001	***
SPAD at heading	P1 × P2	54.05	P2 × P1	55.33	−1.28	−3.40	<0.001	***
SPAD at heading	P1 × P3	51.87	P3 × P1	51.80	0.07	0.18	0.858	ns
SPAD at flowering	P1 × P2	54.48	P2 × P1	55.15	−0.67	−1.51	0.131	ns
SPAD at flowering	P1 × P3	54.06	P3 × P1	54.00	0.07	0.15	0.881	ns
Plant height (cm)	P1 × P2	61.98	P2 × P1	60.17	1.81	1.80	0.073	ns
Plant height (cm)	P1 × P3	61.40	P3 × P1	58.92	2.48	2.73	0.007	**
Spike length (cm)	P1 × P2	9.35	P2 × P1	9.21	0.14	1.12	0.262	ns
Spike length (cm)	P1 × P3	8.78	P3 × P1	8.68	0.10	0.74	0.462	ns
Spikelets per spike	P1 × P2	14.04	P2 × P1	13.85	0.19	0.96	0.339	ns
Spikelets per spike	P1 × P3	12.79	P3 × P1	12.31	0.48	2.58	0.010	*
Grains per spike	P1 × P2	29.97	P2 × P1	26.33	3.64	3.05	0.002	**
Grains per spike	P1 × P3	26.82	P3 × P1	23.94	2.88	2.68	0.008	**
Grain weight per spike (g)	P1 × P2	0.58	P2 × P1	0.50	0.08	2.93	0.004	**
Grain weight per spike (g)	P1 × P3	0.76	P3 × P1	0.71	0.05	1.35	0.177	ns
Grain yield per plant (g)	P1 × P2	0.65	P2 × P1	0.55	0.10	2.95	0.003	**
Grain yield per plant (g)	P1 × P3	0.82	P3 × P1	0.77	0.05	1.22	0.224	ns
Biomass per plant (g)	P1 × P2	3.64	P2 × P1	2.96	0.68	5.15	<0.001	***
Biomass per plant (g)	P1 × P3	3.79	P3 × P1	3.29	0.50	3.34	<0.001	***
Harvest index (%)	P1 × P2	15.01	P2 × P1	15.37	−0.36	−0.59	0.557	ns
Harvest index (%)	P1 × P3	18.21	P3 × P1	19.55	−1.34	−1.81	0.071	ns

* *p* < 0.001; ** *p* < 0.01; *** *p* < 0.05; ns, not significant.

**Table 5:** Percentage of favourable transgressive F_2_ individuals for each trait in the four crosses. Favourable transgression is defined as exceeding the higher parent for the physiological, spike and yield traits, and as falling below the lower parent (earlier or shorter) for days to heading, days to flowering and plant height. Full per-cross parameters (F_2_ mean, SD, CV%, range and counts) are given in Supplementary Tables S1–S4.

Trait (Favourable Direction)	P1 × P2	P2 × P1	P1 × P3	P3 × P1
Days to heading (earlier)	33.0	23.3	73.4	63.3
Days to flowering (earlier)	36.7	24.8	76.3	70.1
SPAD at heading	13.8	27.9	34.8	31.9
SPAD at flowering	30.3	41.7	48.3	44.9
Plant height (shorter)	43.6	51.6	57.5	71.0
Spike length	51.8	40.6	64.3	57.5
Spikelets per spike	71.1	69.4	49.3	37.7
Grains per spike	66.1	50.2	57.5	44.0
Grain weight per spike	49.1	36.1	72.0	63.3
Grain yield per plant	46.3	32.0	57.5	58.0
Biomass per plant	56.0	33.8	63.3	47.3
Harvest index	40.8	37.9	63.3	62.8

Values are percentages of the F_2_ individuals of each cross. *n* = 218 (P1 × P2), 219 (P2 × P1), 207 (P1 × P3), 207 (P3 × P1). P1 = Adana-99, P2 = Alada, P3 = Lucilla.

**Table 6:** Direct effects, total indirect effects and total correlation with grain yield per plant from path-coefficient analysis.

Trait	Direct Effect	Total Indirect	r with GY
Days to heading	0.007	−0.476	−0.469
Days to flowering	−0.005	−0.232	−0.237
SPAD at heading	−0.014	−0.120	−0.134
SPAD at flowering	−0.016	0.038	0.022
Plant height (cm)	0.018	0.502	0.520
Spike length (cm)	−0.088	0.390	0.302
Spikelets per spike	−0.016	0.292	0.275
Grains per spike	0.034	0.646	0.680
Grain weight per spike (g)	0.779	0.139	0.918
Biomass per plant (g)	0.328	0.279	0.606

Residual effect = 0.292.

**Table 7:** Structure matrix of the canonical discriminant analysis: correlations between each trait and the first two canonical functions, and the dominant function for each trait.

Trait	LD1	LD2	Dominant Axis
Days to heading	−0.742	−0.001	LD1
Days to flowering	−0.678	0.015	LD1
SPAD at heading	−0.529	0.091	LD1
SPAD at flowering	−0.138	0.075	LD1
Plant height (cm)	−0.047	−0.371	LD2
Spike length (cm)	−0.287	−0.239	LD1
Spikelets per spike	−0.475	−0.411	LD1
Grains per spike	−0.144	−0.501	LD2
Grain weight per spike (g)	0.449	−0.175	LD1
Grain yield per plant (g)	0.385	−0.193	LD1
Biomass per plant (g)	0.161	−0.615	LD2
Harvest index (%)	0.365	0.316	LD1

LD1, first canonical function (86.8% of the between-cross divergence); LD2, second canonical function (10.6%).

**Table 8:** Selection differentials for the MGIDI-selected F_2_ individuals (15% selection intensity; 128 of 851 plants). Xo, population mean; Xs, mean of the selected group; SD%, selection differential).

**Trait**	**Xo**	**Xs**	**SD%**
Days to heading (d)	68.06	64.25	−5.6
Days to flowering (d)	77.56	75.96	−2.1
SPAD at heading	53.30	56.30	+5.6
SPAD at flowering	54.43	59.46	+9.2
Plant height (cm)	60.63	61.62	+1.6
Spike length (cm)	9.01	9.55	+6.0
Spikelets per spike	13.27	13.98	+5.4
Grains per spike	26.80	33.61	+25.4
Grain weight per spike (g)	0.63	0.93	+47.1
Grain yield per plant (g)	0.70	1.05	+51.4
Biomass per plant (g)	3.41	4.36	+27.6
Harvest index (%)	16.98	19.99	+17.7

## Data Availability

The single-plant phenotypic dataset analysed in this study is available from the corresponding author.

## Supplementary Materials

The following are available online: https://www.mdpi.com/article/10.3390/plants15182780/s1. Table S1. Transgressive segregation and variation parameters for the P1 × P2 cross (Adana-99 × Alada; *n* = 218); Table S2. Transgressive segregation and variation parameters for the P2 × P1 cross (Alada × Adana-99; *n* = 219); Table S3. Transgressive segregation and variation parameters for the P1 × P3 cross (Adana-99 × Lucilla; *n* = 207); Table S4. Transgressive segregation and variation parameters for the P3 × P1 cross (Lucilla × Adana-99; *n* = 207).
