# Supplementary material for: Transgressive Segregation, Reciprocal Effects and Multi-Trait Selection in an F2 Bread Wheat Diallel Under Speed Breeding
Source: Plants (Basel). 2026 Sep 10;15(18):2780. doi: 10.3390/plants15182780 (PMC13610667; doi:10.3390/plants15182780)
Supplement: Supplementary file 1 [file plants-15-02780-s001.zip › plants-4508518-supplementary.pdf]

Supplementary Material

Transgressive Segregation, Reciprocal Effects and Multi-Trait (MGIDI) Selection in an F2 Bread Wheat Population Evaluated under Speed-Breeding Conditions

Table S1. Transgressive segregation and variation parameters for the P1 × P2 cross (Adana-99 × Alada; n = 218).

Trait	LP mean	HP mean	F2 mean	F2 SD	F2 min	F2 max	Range	CV %	< LP	> HP	Trans. n	Trans. %
Days to heading (earlier)	68	75.33	70.24	4.77	61	85	24	6.80	72	26	72	33.03
Days to flowering (earlier)	74.67	82	76.65	4.69	68	89	21	6.12	80	32	80	36.7
SPAD at heading	50.67	58.18	54.05	3.59	43.10	63.80	20.70	6.64	39	30	30	13.76
SPAD at flowering	51.38	55.98	54.48	4.12	44.80	67.60	22.80	7.57	46	66	66	30.28
Plant height, cm (shorter)	60.68	69.45	61.98	9.97	36	87	51	16.09	95	55	95	43.58
Spike length (cm)	8.67	9.83	9.35	1.35	6	12	6	14.49	61	113	113	51.83
Spikelets per spike	12.33	13.67	14.04	2.05	8	20	12	14.60	63	155	155	71.1
Grains per spike	24.28	25.33	29.97	12.14	2	64	62	40.51	64	144	144	66.06
Grain weight per spike (g)	0.35	0.56	0.58	0.30	0.03	1.59	1.56	51.53	49	107	107	49.08
Grain yield per plant (g)	0.44	0.68	0.65	0.37	0.03	2.69	2.66	57.56	68	101	101	46.33
Biomass per plant (g)	2.90	3.23	3.64	1.43	1.01	11.35	10.34	39.20	71	122	122	55.96
Harvest index (%)	12.74	16.91	15.01	6.34	1.12	30	28.88	42.27	78	89	89	40.83

LP, lower-parent mean; HP, higher-parent mean; CV, coefficient of variation; < LP, number of F2 individuals below the lower parent; > HP, number above the higher parent; Trans. n / Trans. %, number and percentage of favourable transgressive individuals (below the lower parent for the traits marked "earlier/shorter", otherwise above the higher parent).

Table S2. Transgressive segregation and variation parameters for the P2 × P1 cross (Alada × Adana-99; n = 219).

Trait	LP mean	HP mean	F2 mean	F2 SD	F2 min	F2 max	Range	CV %	< LP	> HP	Trans. n	Trans. %
Days to heading (earlier)	68	75.33	70.88	4.30	61	85	24	6.07	51	32	51	23.29
Days to flowering (earlier)	74.67	82	77.59	4.31	68	90	22	5.55	54	36	54	24.77
SPAD at heading	50.67	58.18	55.33	4.26	44.60	66.60	22	7.69	30	61	61	27.85
SPAD at flowering	51.38	55.98	55.15	5.07	31.10	67.90	36.80	9.20	43	91	91	41.74
Plant height, cm (shorter)	60.68	69.45	60.17	11.07	37	87	50	18.40	113	46	113	51.6
Spike length (cm)	8.67	9.83	9.21	1.31	5	12	7	14.20	64	89	89	40.64
Spikelets per spike	12.33	13.67	13.85	2.05	8	18	10	14.77	67	152	152	69.41
Grains per spike	24.28	25.33	26.33	12.77	2	62	60	48.49	103	110	110	50.23
Grain weight per spike (g)	0.35	0.56	0.50	0.29	0.01	1.23	1.22	57.55	74	79	79	36.07
Grain yield per plant (g)	0.44	0.68	0.55	0.34	0.01	1.88	1.87	61.36	96	70	70	31.96
Biomass per plant (g)	2.90	3.23	2.96	1.34	0.55	8.73	8.18	45.21	121	74	74	33.79
Harvest index (%)	12.74	16.91	15.37	6.46	0.88	31.94	31.07	42.07	78	83	83	37.9

LP, lower-parent mean; HP, higher-parent mean; CV, coefficient of variation; < LP, number of F2 individuals below the lower parent; > HP, number above the higher parent; Trans. n / Trans. %, number and percentage of favourable transgressive individuals (below the lower parent for the traits marked "earlier/shorter", otherwise above the higher parent).

Table S3. Transgressive segregation and variation parameters for the P1 × P3 cross (Adana-99 × Lucilla; n = 207).

Trait	LP mean	HP mean	F2 mean	F2 SD	F2 min	F2 max	Range	CV %	< LP	> HP	Trans. n	Trans. %
Days to heading (earlier)	67	68	64.97	4.05	57	77	20	6.24	152	40	152	73.43
Days to flowering (earlier)	74.17	74.67	72.22	4.43	60	84	24	6.13	158	49	158	76.33
SPAD at heading	50.67	53.40	51.87	3.97	40.70	61.40	20.70	7.66	79	72	72	34.78
SPAD at flowering	51.38	53.77	54.06	4.53	43.60	68	24.40	8.38	55	100	100	48.31
Plant height, cm (shorter)	64.62	69.45	61.40	9.19	38	82	44	14.96	119	39	119	57.49
Spike length (cm)	8	8.67	8.78	1.32	4	12	8	15.05	27	133	133	64.25
Spikelets per spike	12.33	12.33	12.79	1.93	6	18	12	15.07	105	102	102	49.28

Trait	LP mean	HP mean	F2 mean	F2 SD	F2 min	F2 max	Range	CV %	< LP	> HP	Trans. n	Trans. %
Grains per spike	23.44	25.33	26.82	11.12	1	55	54	41.47	77	119	119	57.49
Grain weight per spike (g)	0.51	0.56	0.76	0.33	0.02	1.74	1.72	44.27	49	149	149	71.98
Grain yield per plant (g)	0.57	0.68	0.82	0.40	0.02	1.89	1.87	48.73	56	119	119	57.49
Biomass per plant (g)	2.76	3.23	3.79	1.54	0.57	9.04	8.47	40.67	57	131	131	63.29
Harvest index (%)	16.91	16.97	18.21	6.38	0.49	40.36	39.87	35.02	75	131	131	63.29

LP, lower-parent mean; HP, higher-parent mean; CV, coefficient of variation; < LP, number of F2 individuals below the lower parent; > HP, number above the higher parent; Trans. n / Trans. %, number and percentage of favourable transgressive individuals (below the lower parent for the traits marked "earlier/shorter", otherwise above the higher parent).

Table S4. Transgressive segregation and variation parameters for the P3 × P1 cross (Lucilla × Adana-99; n = 207).

Trait	LP mean	HP mean	F2 mean	F2 SD	F2 min	F2 max	Range	CV %	< LP	> HP	Trans. n	Trans. %
Days to heading (earlier)	67	68	65.86	4.81	58	84	26	7.31	131	49	131	63.29
Days to flowering (earlier)	74.17	74.67	72.71	5.03	61	89	28	6.91	145	62	145	70.05
SPAD at heading	50.67	53.40	51.80	3.75	40.40	63.50	23.10	7.25	82	66	66	31.88
SPAD at flowering	51.38	53.77	54	4.35	43.50	67.50	24	8.06	55	93	93	44.93
Plant height, cm (shorter)	64.62	69.45	58.92	9.33	35	78	43	15.84	147	32	147	71.01
Spike length (cm)	8	8.67	8.68	1.35	5	12	7	15.51	38	119	119	57.49
Spikelets per spike	12.33	12.33	12.31	1.89	6	18	12	15.32	129	78	78	37.68
Grains per spike	23.44	25.33	23.94	10.79	1	53	52	45.07	101	91	91	43.96
Grain weight per spike (g)	0.51	0.56	0.71	0.35	0.01	1.74	1.73	49.71	64	131	131	63.29
Grain yield per plant (g)	0.57	0.68	0.77	0.42	0.01	2.20	2.19	54.01	69	120	120	57.97
Biomass per plant (g)	2.76	3.23	3.29	1.54	0.40	11.37	10.97	46.78	84	98	98	47.34
Harvest index (%)	16.91	16.97	19.55	8.48	1.23	40.19	38.95	43.41	77	130	130	62.8

LP, lower-parent mean; HP, higher-parent mean; CV, coefficient of variation; < LP, number of F2 individuals below the lower parent; > HP, number above the higher parent; Trans. n / Trans. %, number and percentage of favourable transgressive individuals (below the lower parent for the traits marked "earlier/shorter", otherwise above the higher parent).
